# RNA-Seq Analysis and Candidate Gene Mining of *Gossypium hirsutum* Stressed by *Verticillium dahliae* Cultured at Different Temperatures

**DOI:** 10.3390/plants13192688

**Published:** 2024-09-25

**Authors:** Ni Yang, Zhaolong Gong, Yajun Liang, Shiwei Geng, Fenglei Sun, Xueyuan Li, Shuaishuai Qian, Chengxia Lai, Mayila Yusuyin, Junduo Wang, Juyun Zheng

**Affiliations:** Research Institute of Economic Crops, Xinjiang Academy of Agricultural Sciences, Urumqi 830091, China; yangni157@163.com (N.Y.); g15981775091@163.com (Z.G.); 13999966149@163.com (Y.L.); gengshiwei20201231@163.com (S.G.); xjsunfenglei@163.com (F.S.); xjmh2338@163.com (X.L.); 17509977905@163.com (S.Q.); lchxia2001@163.com (C.L.); mayila324@163.com (M.Y.)

**Keywords:** *G. hirsutum*, temperature, *V. dahliae*, RNA-seq, candidate genes

## Abstract

The occurrence and spread of *Verticillium dahliae* (*V. dahliae*) in cotton depends on the combined effects of pathogens, host plants, and the environment, among which temperature is one of the most important environmental factors. Studying how temperature impacts the occurrence of *V. dahliae* in cotton and the mechanisms governing host defense responses is crucial for disease prevention and control. Understanding the dual effects of temperature on both pathogens and hosts can provide valuable insights for developing effective strategies to manage this destructive fungal infection in cotton. This study was based on the deciduous *V. dahliae* Vd-3. Through cultivation at different temperatures, Vd-3 formed the most microsclerotia and had the largest colony diameter at 25 °C. Endospore toxins were extracted, and 48 h was determined to be the best pathogenic time point for endotoxins to infect cotton leaves through a chlorophyll fluorescence imaging system and phenotypic evaluation. Transcriptome sequencing was performed on cotton leaves infected with Vd-3 endotoxins for 48 h at different culture temperatures. A total of 34,955 differentially expressed genes (DEGs) were identified between each temperature and CK (no pathogen inoculation), including 17,422 common DEGs. The results of the enrichment analysis revealed that all the DEGs were involved mainly in photosynthesis and sugar metabolism. Among the 34,955 DEGs, genes in the biosynthesis and signal transduction pathways of jasmonic acid (JA), salicylic acid (SA), and ethylene (ET) were identified, and their expression patterns were determined. A total of 5652 unique DEGs were clustered into six clusters using the k-means clustering algorithm, and the functions and main transcription factors (TFs) of each cluster were subsequently annotated. In addition, we constructed a gene regulatory network via weighted correlation network analysis (WGCNA) and identified twelve key genes related to cotton defense against *V. dahliae* at different temperatures, including four genes encoding transcription factors. These findings provide a theoretical foundation for investigating temperature regulation in *V. dahliae* infecting cotton and introduce novel genetic resources for enhancing resistance to this disease in cotton plants.

## 1. Introduction

Temperature is an important environmental factor in the growth and pathogenicity of plant pathogens [1]. Many reports have described the inhibitory effects of high or low temperatures on the growth and infection of pathogens [2]. *Verticillium dahliae* is extremely sensitive to temperature and mutates due to culture conditions, temperature, and other environmental factors [3]. Its morphological variation is manifested mainly in changes in colony morphology and the increase or loss in microsclerotia and sclerotia [4]. The suitable temperature for the growth of *V. dahliae* on cotton is 22–25 °C, with high conidia and toxin production and the greatest pathogenicity [5]. In early July, the temperature in the cotton-producing area in northern Xinjiang, China, is mostly between 25 and 30 °C, which creates favorable conditions for the survival of *V. dahliae* [6]. An appropriate temperature can accelerate the infection and reproduction of pathogens. When the temperature is above 35 °C, the pathogen stops growing and reproducing and will not infect plants, and thus high-temperature latent symptoms will occur. High-temperature hidden syndrome is an extreme manifestation of *V. dahliae* infection of cotton in the field. It is an adaptive mechanism of *V. dahliae* to cope with adverse environments [7]. The longer the high temperature is, the lower the activity of the pathogen, the weaker the infection ability, and the disease cannot spread. The continuous high temperatures in the Xinjiang cotton-producing area in July and August every year have a very obvious inhibitory effect on the occurrence of the disease. When the temperature decreases, the pathogen resumes growth, and the incidence rate increases [8]. This phenomenon has a great impact on the growth of cotton fields and has severely hindered the progress of cotton resistance research.

The transcriptome has been widely used to study plant disease resistance. Transcriptome data analysis revealed that the expression of the *OsHLP1* gene in rice was significantly upregulated in response to rice blast infection. *OsHLP1*-overexpressing plants exhibited significantly increased resistance to rice blast [9]. Significant differences in gene expression were observed between two sesame varieties, suggesting that the upregulation of differentially expressed genes (DEGs) among the disease resistance (R) genes may increase resistance. Moreover, combined with the phenotypic observations of sesame leaves inoculated at different time points, a comparative transcriptome analysis of Corynespora leaf spot from the two sesame varieties revealed that 12 h post-inoculation (hpi) was the key time point leading to the difference in resistance between the two materials. A weighted correlation network analysis (WGCNA) identified two modules significantly associated with disease resistance and identified ten key candidate genes [10]. Through an RNA-seq analysis of the metabolite components of eggplants with high resistance and high sensitivity to *V. dahliae*, after inoculation with *V. dahliae*, the highly resistant material presented greater polyphenol oxidase and superoxide dismutase activities than did the highly resistant material, and 4017 DEGs were identified. A coexpression analysis identified 13 transcription factors as key genes related to the eggplant defense response [11]. Through an RNA-seq analysis of the highly *V. dahliae*-susceptible cotton variety Junmian 1 and the highly resistant cotton variety Liaomian 38, most DEGs were annotated to resistance-related pathways. Resistance gene analogs (RGAs) have also been identified, and their roles in enhancing *V. dahliae* resistance in Liaomian 38 were analyzed [12]. A comparative RNA-seq analysis of the upland cotton stems of the *V. dahliae* varieties Jimian 11 and Zhongzhimian 2 revealed 8330 DEGs and 383 miRNAs, and 31 differentially expressed miRNA-mRNA pairs were identified [13].

Increases in global temperature and extreme temperature fluctuations are important trends in future climate change [14]. Understanding the dual impacts of temperature on *V. dahliae* occurrence in cotton and the host response is crucial for understanding the pathogenesis of this disease. Therefore, analyzing how various temperatures affect the occurrence of *V. dahliae* in cotton, studying the mechanism regulating host defense, and revealing the dual effects of temperature on pathogens and hosts are essential for achieving environmentally friendly disease prevention and control and temperature regulation. This study is based on defoliating *V. dahliae* (Vd-3). Endotoxins were extracted at different culture temperatures, the best pathogenic time point for endotoxins to infect cotton leaves was determined using a chlorophyll fluorescence imaging system, and the materials obtained at this time were subjected to RNA-seq. Through a differential expression analysis, cluster analysis, enrichment analysis, expression pattern analysis of hormone biosynthesis and signal transduction genes, WGCNA, and qRT-PCR, the key genes that induce the cotton defense system and the regulatory pathways related to defense were further characterized, revealing the mechanism of cotton resistance to temperature sensitivity. These results are highly important for research on cotton resistance to *V. dahliae* and genetic engineering, and provide theoretical support for the effective prevention and control of *V. dahliae* and for temperature regulation research.

## 2. Results

### 2.1. Phenotypes of Vd-3 Inoculated at Different Times and Temperatures

**Figure 1:**
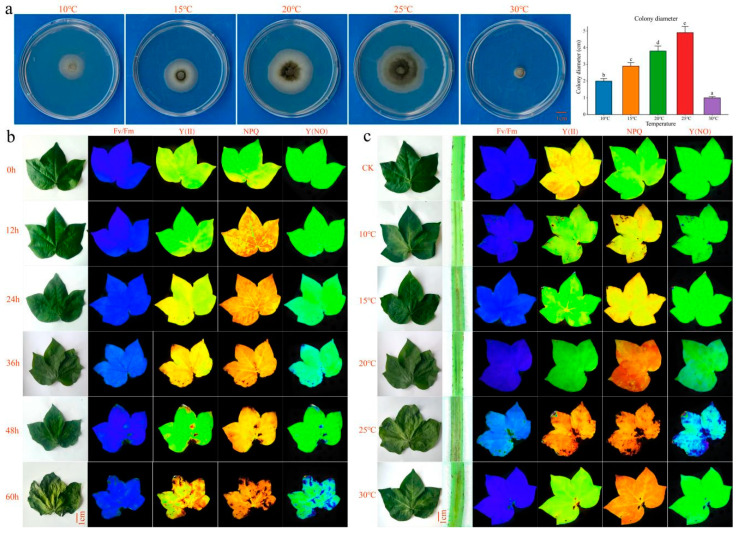
(**a**) Phenotype and colony diameter of Vd-3 colonies after 14 days of culture at different temperatures, bar = 1 cm. Different letters indicate the significance level of difference in colony diameter at different temperatures (*p* < 0.05). (**b**) Phenotype and chlorophyll fluorescence imaging of cotton leaves infected with the spore toxin protein at different times under normal conditions; bar = 1 cm. (**c**) Phenotype and chlorophyll fluorescence imaging of cotton leaves infected with spore toxin protein at different temperatures for 48 h; bar = 1 cm.

Since different temperatures can affect the growth of hyphae, they can also affect the spore production, spore shape, size, etc., of the pathogen, ultimately affecting the germination, infection, and spread of the pathogen. To this end, the Verticillium wilt pathogen Vd-3 was inoculated on PDA solid media and cultured for 14 days. At 25 °C, the greatest number of microsclerotia formed, and the largest colony diameter was observed (Figure 1a). Phenotypic chlorophyll fluorescence imaging of leaves at different time points (0 h, 12 h, 24 h, 36 h, 48 h, and 60 h) revealed that the leaves wilted at 48 h (Figure 1b). Using 48 h as the key period of infection, the degree of wilting of the leaves of the inoculated plants increased with increasing culture temperature. At 25 °C, the disease was most severe, the vascular bundles browned, and Fv/Fm, Y(II), NPQ, and Y(NO) all reached their maximum values (Figure 1c). Therefore, RNA-seq was performed on samples of Verticillium wilt-infected cotton cultured at different temperatures for 48 h to analyze the regulatory pathways and key genes of Verticillium wilt-infected cotton cultured at different temperatures.

### 2.2. RNA-Seq Data Analysis

**Figure 2:**
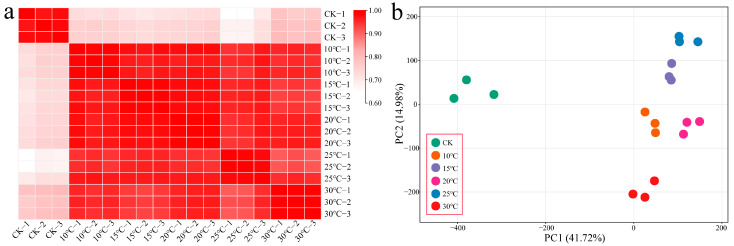
(**a**) Correlation analysis of RNA-seq data from cotton leaves infected with the spore toxin protein at different temperatures. (**b**) PCA of RNA-seq data from cotton leaves infected with the spore toxin protein at different temperatures.

This study generated 124.3 Gb of clean data from 18 samples of cotton leaves infected with Vd-3 at various culture temperatures (10 °C, 15 °C, 20 °C, 25 °C, and 30 °C). Each sample had 6.29 Gb of clean data, with a Q30 base percentage exceeding 92.98% and an alignment rate surpassing 97.86% (Table S2). By calculating the Pearson correlation coefficient between three biological replicates of the same sample, a correlation exceeding 0.98 was observed (Figure 2a). The PCA results revealed that the biological replicate samples clustered together, suggesting the high reliability and repeatability of the transcriptome data (Figure 2b).

### 2.3. Differential Expression Analysis

**Figure 3:**
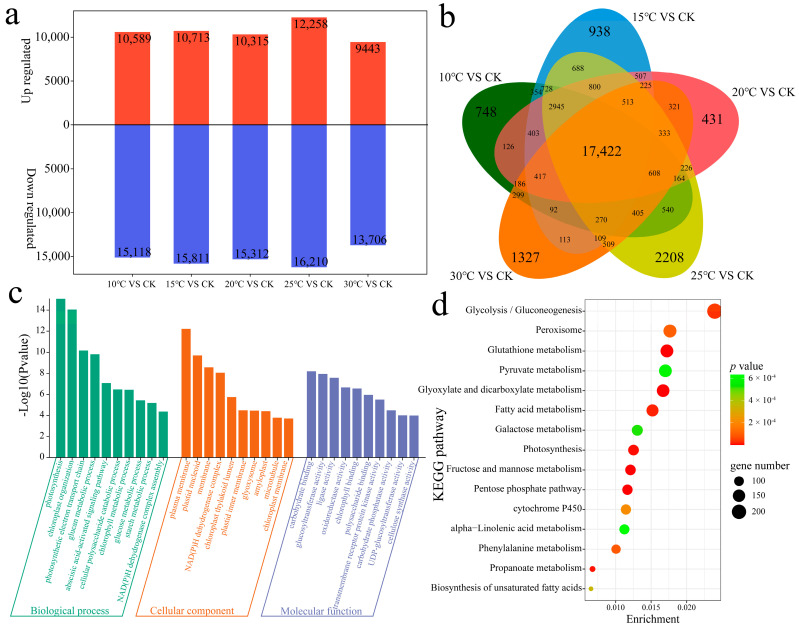
(**a**) Numbers of upregulated and downregulated DEGs at different temperatures. (**b**) Numbers of common and unique DEGs at different temperatures. (**c**) GO enrichment analysis of DEGs. (**d**) KEGG enrichment analysis of DEGs.

DEGs between samples cultivated each temperature, and the CK were identified to investigate the regulatory patterns of *V. dahliae* infection in cotton cultivated at different temperatures (Figure 3a). At 10 °C, a total of 25,707 DEGs were identified: 10,589 were upregulated, 15,118 were downregulated, and 748 were unique DEGs. At 15 °C, a total of 26,524 DEGs were identified: 10,713 were upregulated, 15,811 were downregulated, and 938 were unique DEGs. At 20 °C, a total of 25,627 DEGs were identified: 10,315 were upregulated, 15,312 were downregulated, and 431 were unique DEGs. At 25 °C, a total of 28,468 DEGs were identified: 12,258 were upregulated, 16,210 were downregulated, and 2208 were unique DEGs. At 30 °C, a total of 23,149 DEGs were identified, including 9443 upregulated, 13,706 downregulated, and 1327 unique DEGs. A total of 34,955 DEGs were identified under all the temperature conditions, including 17,422 common DEGs (Figure 3b).

Annotation of differentially expressed genes helps us to further understand the functions of genes. We performed GO and KEGG enrichment analyses on 34,955 DEGs. GO enrichment analysis revealed that the biological process terms included photosynthesis, chloroplast organization, the photosynthetic electron transport chain, the glucan metabolic process, the abscisic acid-activated signaling pathway, the cellular polysaccharide catabolic process, the chlorophyll metabolic process, the glucose metabolic process, and the starch metabolic process. The main molecular functions included carbohydrate binding, glucosyltransferase activity, ligase activity, oxidoreductase activity, chlorophyll binding, polysaccharide binding, transmembrane binding, membrane receptor protein kinase activity, carbohydrate phosphatase activity, UDP-glucosyltransferase activity, and cellulose synthase activity (Figure 3c). Enriched KEGG pathways were associated mainly with glyoxylate and dicarboxylate metabolism, the pentose phosphate pathway, fructose and mannose metabolism, glutathione metabolism, photosynthesis, propanoate metabolism, fatty acid metabolism, glycolysis/gluconeogenesis, phenylalanine metabolism, peroxisome metabolism, cytochrome P450, unsaturated fatty acid biosynthesis, galactose metabolism, pyruvate metabolism, and alpha-linolenic acid metabolism (Figure 3d).

**Figure 4:**
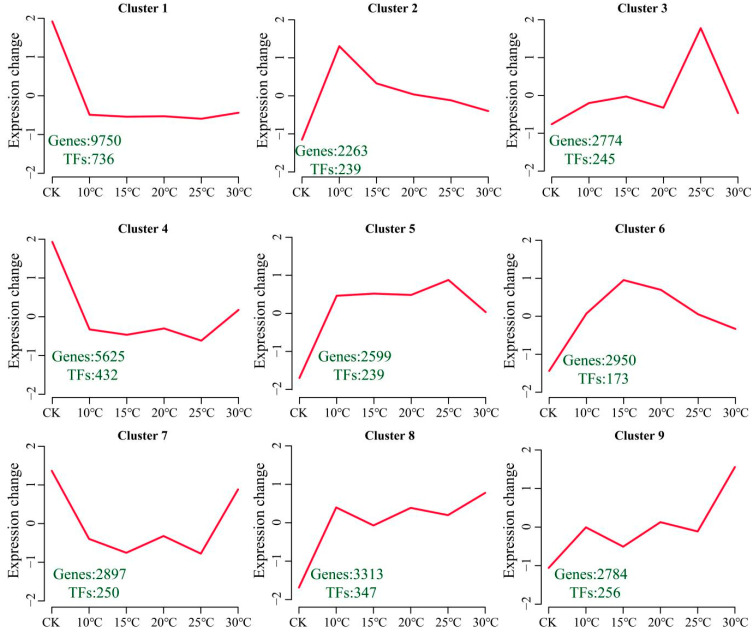
Line graph of the cluster analysis of DEGs. The green numbers represent the numbers of DEGs and TFs in each cluster.

The functional changes in genes under *V. dahliae* stress in cotton cultured at different temperatures were investigated by clustering the 34,955 DEGs into nine clusters using the k-means clustering algorithm, and the number of transcription factors (TFs) in each cluster was determined (Figure 4). Cluster 1 presented the highest expression level under CK conditions, with 736 TFs among the 9750 DEGs. Cluster 2 presented the highest expression level at 10 °C, with 239 TFs among the 2263 DEGs. Cluster 3 presented the highest expression level at 25 °C, with 245 TFs among the 2274 DEGs. Cluster 4 presented the highest expression level under CK and 30 °C conditions, with 432 TFs among the 5625 DEGs. Cluster 5 presented the lowest expression level under CK conditions, with 239 TFs among the 2599 DEGs. Cluster 6 presented the lowest expression level at 15 °C, with 173 TFs among the 2950 DEGs. Cluster 7 presented the highest expression level under CK and 30 °C conditions and contained 250 TFs among 2897 DEGs. Cluster 8 presented the lowest expression level under CK conditions and included 347 TFs among the 3313 DEGs. Cluster 9 presented the lowest expression level under CK conditions and the highest expression level at 30 °C and contained 256 TFs among the 2784 DEGs.

**Figure 5:**
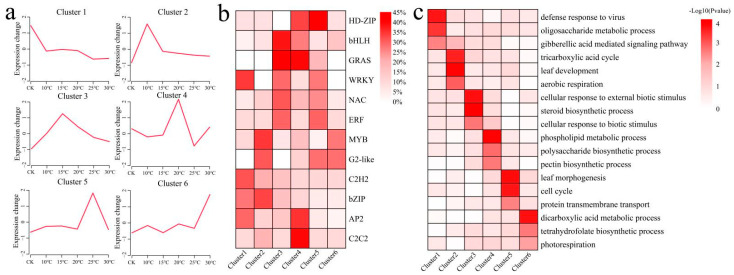
(**a**) Line graph of the cluster analysis of unique DEGs. (**b**) Heatmap of TF proportions in each cluster. (**c**) Heatmap of the KEGG enrichment analysis results for each cluster.

We clustered the 5652 unique DEGs into six clusters using the k-means clustering algorithm and then annotated the functions and main TFs of each cluster to further understand the functions of the unique DEGs (Figure 5). Cluster 1 genes, which included mainly the WRKY, C2H2, bZIP, and AP2 transcription factors, were highly expressed under CK conditions and annotated in the defense response to virus, oligosaccharide metabolic process, and gibberellic acid-mediated signaling pathways. Cluster 2 genes, which included mainly MYB, G2-like, and bZIP transcription factors, were highly expressed at 10 °C and annotated in the tricarboxylic acid cycle, leaf development, and aerobic respiration. Cluster 3 genes, which included bHLH, GRAS, WRKY, NAC, and ERF transcription factors, were highly expressed at 15 °C and annotated as being involved in the cellular response to external biotic stimuli, the steroid biosynthetic process, and the cellular response to biotic stimuli. Cluster 4 genes were highly expressed at 20 °C, enriched in phospholipid metabolic processes, polysaccharide biosynthetic processes, and pectin biosynthetic processes, and included mainly HD-ZIP, bHLH, GRAS, AP2, and C2C2 transcription factors. Cluster 5 genes were highly expressed at 25 °C, annotated as being involved in leaf morphogenesis, the cell cycle, and protein transmembrane transport, and included mainly HD-ZIP, ERF, and G2-like transcription factors. Cluster 6 genes were highly expressed at 30 °C, annotated in dicarboxylic acid metabolic processes, tetrahydrofolate biosynthetic processes, and photorespiration, and included mainly MYB and G2-like transcription factors (Figure 5).

### 2.4. Analysis of Patterns of Gene Expression in the JA, SA, and ET Pathways

**Figure 6:**
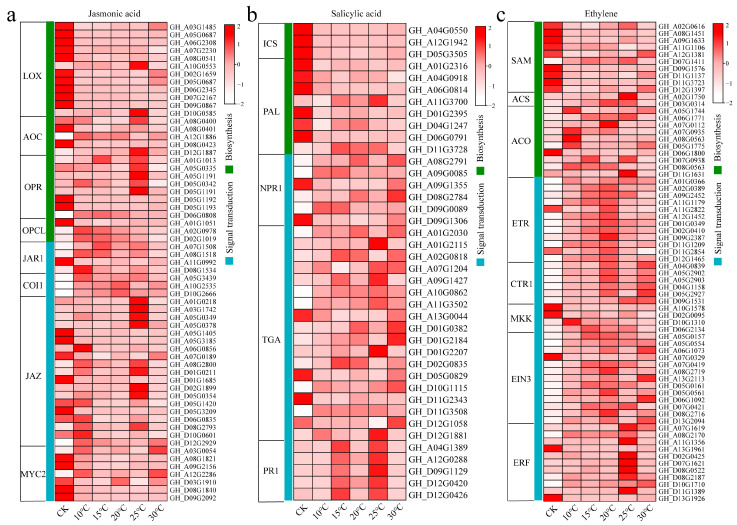
(**a**) Calorimetry of JA biosynthesis and signaling-related DEGs. (**b**) Calorimetry of SA biosynthesis and signaling-related DEGs. (**c**) Calorimetry of ET biosynthesis and signaling-related DEGs.

JA, SA, and ET are believed to play important roles in the response of cotton to *V. dahliae* stress. To this end, we identified 184 DEGs in the JA, SA, and ET biosynthesis and signal transduction pathways (Figure 6). In the JA biosynthesis pathway, LOX genes were mostly expressed at the highest level under CK conditions, two AOC genes (*GH_A08G0400* and *GH_D12G1887*) were expressed at the highest level at 25 °C, and most OPR genes were expressed at the highest level at 25 °C (Figure 6a). In the JA signal transduction pathway, the expression levels of JAR1 and COI1 increased with increasing temperature. Some JAZ genes were expressed at the highest level under CK conditions, and some were expressed at the highest level at 25 °C. Two MYC2 genes (*GH_A03G0054* and *GH_A12G2286*) were expressed at the highest level at 30 °C, and the remaining genes were expressed at the highest level under CK conditions. In the SA biosynthesis pathway, only two PAL genes (*GH_A11G3700* and *GH_D11G3728*) were expressed at the highest level at 25 °C, and the remaining ICS and PAL genes were expressed at the highest level under CK conditions (Figure 6b). In the SA signal transduction pathway, two NPR1 genes (*GH_A09G1355* and *GH_D09G1306*) were expressed at the highest level under CK conditions, and the expression levels of the remaining NPR1 genes increased under different temperature conditions. Among the TGA genes, three genes (*GH_A13G0044*, *GH_D05G0829* and *GH_D11G2343*) were expressed at the highest level under CK conditions, and the expression levels of the remaining genes increased under different temperature conditions. The PR1 gene was expressed at the highest level at 15 °C and 25 °C. In the ET biosynthesis pathway, most of the SAM genes were expressed at the highest level under CK conditions, and the ACS genes were expressed at the highest level at 25 °C (Figure 6c). Only one ACO gene (*GH_D06G1800*) was expressed at the highest level under CK conditions, while the expression levels of the other ACO genes increased under different temperature conditions. In the ET signal transduction pathway, most ETR, CTR1, and EIN3 genes were expressed at the highest level at 20 °C, and except for those of *GH_A13G1961* and *GH_D13G1926*, which were expressed at the highest level under CK conditions, the expression levels of the other ERF genes were all highest at 25 °C.

### 2.5. WGCNA

**Figure 7:**
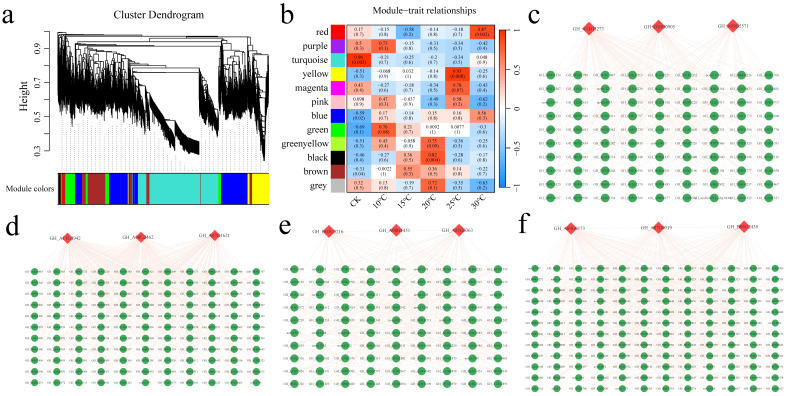
(**a**) WGCNA module hierarchical clustering tree diagram; different modules are represented by different colors. (**b**) Correlation and significance heatmaps between samples and modules. (**c**) Red module gene interaction network diagram. (**d**) Turquoise module gene interaction network diagram. (**e**) Yellow module gene interaction network diagram. (**f**) Black module gene interaction network diagram.

Based on the expression profiles of the 34,955 DEGs, a total of seven coexpression modules were obtained, and different colors were used to represent different modules (Figure 7a). The correlations between the modules and the *V. dahliae* fungus cultured at different temperatures were calculated, and four (red, turquoise, yellow and black) significantly highly correlated modules were identified (r > 0.80, *p* < 0.01) (Figure 7b). For each module, the three genes with the highest connectivity were identified as hub genes, and twelve hub genes were ultimately obtained (Figure 7c–f). The twelve genes included four genes encoding transcription factors (*GH_A11G1273* (bHLH), *GH_A09G2571* (ERF), *GH_A07G1621* (MYB), and *GH_A07G0019* (WRKY)). *GH_D10G0905* encodes an NRT1/PRT family gene, which is an ABA transporter. *GH_A11G1942* encodes a serine/threonine protein phosphatase. *GH_A09G2462* encodes 1-aminocyclopropane-1-carboxylic acid oxidase (ACO), the rate-limiting enzyme regulating ethylene biosynthesis. *GH_D05G3216* encodes cytochrome P450, *GH_A08G1451* encodes S-adenosylmethionine synthase 2, *GH_A05G0363* encodes sucrose synthase, *GH_A09G0173* encodes beta-1,3-galactosyltransferase, and *GH_D06G2430* encodes ADP-ribosylation factor GTPase-activating protein.

### 2.6. qRT-PCR

**Figure 8:**
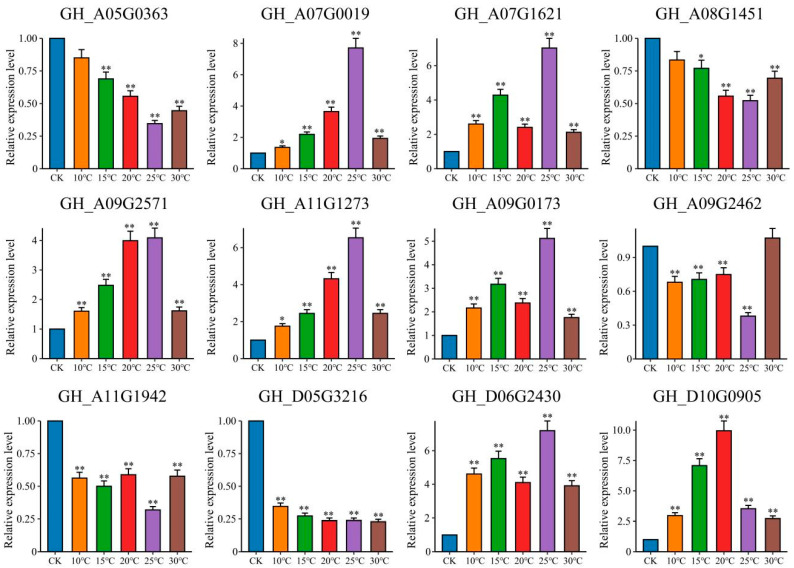
Analysis of the expression patterns of candidate genes under different temperature conditions (error bars represent the means ± SEs of three replicates, * *p* < 0.05 and ** *p* < 0.01).

The expression patterns of the 12 candidate genes after inoculation with *V. dahliae* at different temperatures were detected using qRT-PCR (Figure 8). Compared with those in the CK treatment, the expression levels of five genes (*GH_A05G0363*, *GH_A08G1451*, *GH_A09G2462*, *GH_A11G1942*, and *GH_D05G3216*) decreased significantly after inoculation (Figure 8). Compared with those in the CK treatment, the expression levels of four genes (*GH_A05G0363*, *GH_A08G1451*, *GH_A09G2462*, and *GH_A11G1942*) were the lowest at 25 °C. The expression levels of seven genes (*GH_A07G0019*, *GH_A07G1621*, *GH_A09G0173*, *GH_A09G2571*, *GH_A11G1273*, *GH_D06G2430*, and *GH_D10G0905*) increased significantly after inoculation with *V. dahliae* (fold change > 4). Except for *GH_D10G0905*, the other six genes presented the highest expression levels at 25 °C. The transcriptome data of these 12 genes were significantly correlated with the fold difference in the qRT-PCR data (R = 0.90, *p* < 0.01). These results indicate that the transcriptome sequencing data are reliable (Figure S1).

## 3. Discussion

Temperature is the main factor limiting the occurrence and spread of *V. dahliae*. The optimum temperature for *V. dahliae* in cotton is 25–28 °C. Temperatures below 22 °C or above 33 °C are not conducive to the onset of the disease, and temperatures above 35 °C lead to latent symptoms [6,7]. Temperature is closely related to the growth and reproduction of pathogenic fungi [15]. Temperature not only affects the growth of mycelia, but also affects the spore production, spore shape, size, etc., of pathogens, ultimately affecting their germination, infection, and spread. In addition, different pathogens and even different subspecies of the same pathogen require different suitable temperatures to infect the host. We inoculated the deciduous *V. dahliae* pathogen Vd-3 onto PDA solid culture media and cultured it at 10 °C, 15 °C, 20 °C, 25 °C, and 30 °C. We found that the greatest number of microsclerotia formed at 25 °C, and the largest colony diameter was observed at this temperature. At 25 °C, the cotton plants were the most severely ill, with wilting of the leaves and browning of the vascular bundles. More DEGs (28468) were produced at 25 °C. Our results showed that 25 °C was most suitable for the growth of Vd-3 and resulted in the highest infection efficiency for cotton.

Chloroplasts are among the most dynamic organelles in plant cells. They play an active role in defense responses and are essential for intercellular signaling [16]. Studies have shown that radish leaf curl virus β-satellite infection can destroy the structural and functional integrity of host chloroplasts, leading to the inhibition of photosynthesis and the development of symptoms [17]. N receptor interacting protein 1 (NRIP1) is localized in chloroplasts. When TMV invades, NRIP1 can interact with the N protein and tobacco mosaic virus effector p50 at the same time and is recruited to the cytoplasm and nucleus, thereby mediating resistance to TMV [18]. Silencing the expression of the *rbcS* gene can also mediate resistance to TMV [19]. Our findings revealed that all DEGs were significantly enriched in photosynthesis, chloroplast organization, the photosynthetic electron transport chain, the glucan metabolic process, and the abscisic acid-activated signaling pathway, and many DEGs related to photosynthesis pathways were upregulated. Therefore, photosynthesis is crucial for cotton resistance to *V. dahliae*, and many reports have documented the effects of virus infection on host photosynthesis. Maize dwarf mosaic virus causes a decrease in chloroplast volume and number, a decrease in chloroplast content, and a decrease in the photosynthetic rate [20]. The net photosynthetic rate of tobacco leaves infected with CMV decreases, and the activities of PSI and PSII are inhibited. The pathway for transporting photosynthetic products is the phloem, and the main form of transport is sucrose. Sucrose catabolism plays an important role in plant defense against pathogens [21]. On the one hand, sucrose catabolism can provide carbon skeletons and energy for plant defense responses [22]. For example, defense responses such as cell wall thickening, phytoalexin synthesis, and the accumulation of disease-related proteins require a large amount of sugar [23]. On the other hand, the hexoses (glucose and fructose) produced by sucrose decomposition can also act as signaling molecules to regulate the expression of disease-related genes [24]. Although sugar metabolism can increase plant disease resistance, the hexoses (glucose and fructose) produced by sucrose decomposition can also provide energy and carbon skeletons for the growth of pathogens, thereby causing plant disease. Therefore, sugar metabolism plays both positive and negative roles in the process of plant disease resistance [25]. All DEGs were enriched in the pentose phosphate pathway, fructose and mannose metabolism, glycolysis/gluconeogenesis, and galactose metabolism pathways. Sugars can be used by plants for defense reactions or can be absorbed and utilized by pathogenic bacteria after being decomposed into glucose and fructose. Two enzymes in plants decompose sucrose, namely, sucrose synthase and invertase [26]. SS can decompose sucrose into UDP-glucose and fructose and is one of the key enzymes required for sucrose to enter various metabolic pathways. Among the candidate genes we discovered, *GH_A09G0173* encodes a beta-1,3-galactosyltransferase, and *GH_A05G0363* encodes sucrose synthase. These two genes may be highly important for the resistance of upland cotton to *V. dahliae*.

In the process by which plants resist biological adversity, the salicylic acid-mediated defense response plays a vital role [27]. Studies have shown that the application of exogenous SA or SA analogs to plants can induce the expression of pathogenesis-related proteins in plants, increasing their resistance to pathogens [28]. After pathogens are inoculated into disease-resistant varieties of various crops, such as tobacco, SA accumulates rapidly and in large quantities, and the expression level of pathogenesis-related proteins increases accordingly, triggering a series of downstream immune responses [29]. In the SA biosynthesis pathway, two PAL genes (*GH_A11G3700* and *GH_D11G3728*) were highly expressed at 25 °C, indicating that these two genes play important roles in the dependence of cotton on the SA pathway to improve resistance to *V. dahliae*. Notably, *GH_A09G2462* encodes 1-aminocyclopropane-1-carboxylic acid oxidase (ACO), the rate-limiting enzyme that regulates ethylene biosynthesis. These comprehensive results indicate that hormones, photosynthesis, and sugar metabolism play important roles in the defense of cotton against *V. dahliae*. In the future, the key genes in these regulatory pathways can be functionally verified, and their mechanisms can be analyzed, which are highly important for the resistance of cotton to *V. dahliae*.

TFs are believed to play important roles in the disease resistance of plants. By regulating gene expression in host plants, TFs can affect the degree of infection of plants by pathogens and reduce the harm caused by diseases to plants [30]. For example, some TFs can promote the production of antimicrobial agents in plants and increase the resistance of plants to pathogens, and some TFs can regulate the immune system of plants and improve disease resistance [30]. In addition, some TFs can also regulate the signal transduction pathways of plants, improve the ability of plants to recognize pathogens, and strengthen the disease resistance of plants [31]. For example, *GbERF1-like* has similar expression characteristics to those of *GbERF1* and *GbERF2*. The overexpression of *GbERF1-like* genes in cotton and Arabidopsis can promote the expression of lignin synthesis-related genes and increase resistance to *V. dahliae*. *GbERF1-like* can also interact with the cell wall-related genes pectin methylesterase inhibitory protein *GhPMEI3* and *PMEs* and can regulate the expression of fungus-specific polygalacturonase (*VdPG1*), thereby increasing cotton resistance to *V. dahliae* [32]. The overexpression of the cotton gene *GhMYB4*, which encodes a negative regulator of lignin synthesis, reduces lignin synthesis in vivo, leading to changes in cell wall integrity and the release of more oligogalactosides (OGs), thereby reducing the defense response of cotton to *V. dahliae* [33]. Silencing *GhWRKY70* can lead to the upregulation of JA signaling pathway-related gene expression and the downregulation of SA signaling pathway-related gene expression. The overexpression *GhWRKY70* of transgenic *Arabidopsis thaliana* increased the expression of SA-related genes and decreased the expression of JA response-related genes, thereby reducing the resistance of cotton and *A. thaliana* to *V. dahliae* [34]. *GhWRKY70D13* regulates cotton resistance to *V. dahliae* through the ET and JA signaling pathways [35]. *GhbHLH171* has been found to regulate cotton resistance to *V. dahliae* through the JA signaling pathway [36]. As research has progressed, an increasing number of TFs have been shown to regulate cotton resistance to *V. dahliae*. We also screened four new TFs (*GH_A11G1273* (bHLH), *GH_A09G2571* (ERF), *GH_A07G1621* (MYB), and *GH_A07G0019* (WRKY)), which may play important roles in the response of cotton to *V. dahliae*. In the future, we can focus on cloning these candidate genes, clarifying the regulatory pathways and interaction processes between them, and combining molecular biology and multiomics sequencing to analyze the mechanism of cotton resistance to *V. dahliae* comprehensively.

## 4. Materials and Methods

### 4.1. Plant Materials

*V. dahliae* Vd-3, a fungus causing *V. dahliae* in cotton, was isolated and purified from a diseased cotton field in the cotton-growing area of Changji Prefecture, Xinjiang, China, in October 2018. The pathogenic strain was identified as highly pathogenic and preserved in the laboratory of the Institute of Economic Crops, Xinjiang Academy of Agricultural Sciences. The cotton variety selected in this study was Xinlu Zao 57, which was provided by the Institute of Economic Crops, Xinjiang Academy of Agricultural Sciences. Indoor experiments were conducted at the Xinjiang Academy of Agricultural Sciences in March 2023. The soil and vermiculite were mixed at a mass ratio of 2:1 as a seedling medium and sterilized at 121 °C for 20 min. Full and consistent seeds were selected, disinfected with 5% sodium hypochlorite for 5 min and 75% alcohol for 30 s, rinsed with sterile water three times, and placed in a glass culture dish containing filter paper. The culture dish was placed in an incubator at 24–28 °C for germination. Finally, the germinated seeds were transferred to a 32-well plug tray rich in seedling medium, and the plug tray was moved to a greenhouse at 25–30 °C for seedling cultivation. The light intensity was 4000 lx, and the photoperiod was 16 h/8 h (day/night). In April 2023, *V. dahliae* Vd-3 was inoculated into potato dextrose agar (PDA) medium and cultured at 10 °C, 15 °C, 20 °C, 25 °C, and 30 °C for 14 days. After the diameter of each colony was determined, a sterile punch with a diameter of 5 mm was used to generate a bacterial pellet at the edge of the colony, which was subsequently inoculated into Czapek’s culture medium. The culture was shaken in a shaker at 10 °C, 15 °C, 20 °C, 25 °C, and 30 °C at 130 r/min until the fluorescence value of the fungal mixture was 0.4–0.6 and then filtered with 4 layers of sterile gauze to obtain a conidial suspension of the pathogen. After centrifugation, the precipitate was collected and suspended in 0.05 mol/L phosphate buffer at pH 6.5. The conidia content of the pathogen was detected using a hemocytometer, and the concentration of the suspended conidia was adjusted to 10^7^ conidia/mL. The toxin protein was isolated from the spores of *V. dahliae* Vd-3 grown at 25 °C by ultrahigh-pressure ultrasonic fragmentation. When the cotton seedlings grew 10 true leaves, cotton leaves of the same size and at the same site were selected for infection with spore toxins cultured at different temperatures (10 °C, 15 °C, 20 °C, 25 °C, 30 °C) by the in vitro leaf immersion method, and phenotypic chlorophyll fluorescence imaging of the leaves was performed at different times (0 h, 12 h, 24 h, 36 h, 48 h, and 60 h). The concentration of toxins used in all in vitro leaf toxin immersion tests was 16 μg/mL. Phosphate buffer treatment was used as a control, and the samples were placed in a sterile greenhouse at 25 °C for culture under weak light. Leaves inoculated with bacteria for 48 h at different temperatures (10 °C, 15 °C, 20 °C, 25 °C, 30 °C) were sampled, with 6 biological replicates for each sample (3 for RNA-seq and 3 for qRT-PCR).

### 4.2. Exploration of the Physiological Changes in Cotton Resistance to Vd-3 Virus-Produced Toxins

An EPSON camera system (EPSON, Nagano-ken, Japan) was used to record the phenotypic changes in infected and uninfected cotton seedling leaves, and a stereomicroscope was used to record the changes in the leaf’s anatomical structure. A chlorophyll fluorescence imaging system was used to image detached leaves infected with toxins. After 30 min of dark adaptation, the cotton leaves were placed in a modulated chlorophyll fluorescence imaging system (WALZ product, Effeltrich, Germany, model IMAGING-PAM) to measure the maximum quantum yield (Fv/Fm), actual quantum yield (Y(II)), nonphotochemical quenching (NPQ), quantum yield of nonregulated energy dissipation of photosystem II (Y(NO)), and other chlorophyll fluorescence parameters, and the fluorescence parameters and images were analyzed.

### 4.3. RNA-Seq Library Construction and Sequencing

RNA was extracted using a polysaccharide and polyphenol plant total RNA extraction kit (each sample was guaranteed to be approximately 100 mg), and the extraction process was performed according to the instructions. After RNA extraction, a NanoDrop spectrophotometer (Thermo Fisher Scientific, Waltham, MA, USA) was used to determine the purity (OD260/280), concentration, and whether the nucleic acid absorption peak was normal. The integrity of the RNA was accurately detected with an Agilent 2100 instrument (Agliment, Orange, CA, USA). After each sample was qualified, it was transported on dry ice to Xinjiang Aidesen Biological Co., Ltd. (Urumqi, China) for sequencing. Magnetic beads with oligo(dT) were used to enrich mRNAs by combining A-T complementary pairing with the poly(A) tail of the mRNA. Subsequently, fragmentation buffer was added to shear the mRNA into short fragments. Using the mRNA as a template, random hexamers were used to synthesize the first-strand cDNA, and then buffer, dNTPs, and DNA polymerase I were added to synthesize the second-strand cDNA. The double-stranded cDNA was then purified using AMPure XP beads (Metware, Wuhan, China). The purified double-stranded cDNA was then end-repaired, A-tailed, and connected to sequencing adapters. Then, AMPure XP beads were used for fragment size selection, and PCR enrichment was performed to obtain the final cDNA library. The library was detected using an Agilent 2100 instrument (Agliment, Orange, CA, USA) and Q-PCR. The constructed library was sequenced on the Illumina HiSeq 2500 sequencing (Illumina, San Diego, CA, USA) platform.

### 4.4. RNA-Seq Analysis

After the raw sequence data were obtained, Fastp software (version 0.23.4) was used to remove the adapter sequences and filter out low-quality and N sequences with a ratio greater than 5% to obtain clean reads that could be used for subsequent analysis [37]. HISAT2 was used to align the clean reads to the reference genome of TM-1 (https://www.cottongen.org/species/Gossypium_hirsutum/ZJU-AD1_v2.1, (accesssed on 15 July 2023)) [38,39]. FeatureCounts was used to compare the results for statistics and quantification [40]. Assessing the correlation of biological replicates is crucial in transcriptome sequencing data analysis. This evaluation not only confirms the consistency of biological experimental procedures, but also ensures the credibility of differentially expressed genes and aids in identifying any outliers. The R language PCAtools software (version 2.16) package was used to decompose the expression data (FPKM) of all genes into n principal components to describe the characteristics of the original dataset. PC1 represents the most obvious feature that can be described in the multidimensional data matrix, and PC2 represents the most significant feature that can be described in the data matrix except PC1. The ggplot software package was used for visualization. The unnormalized read count data were used as input data to calculate the *p* value and fold change value with DESeq2 software (version 1.44). A *p* value < 0.05 and a |log2 fold change| > 1 were used as screening criteria to obtain DEGs [41]. Enrichment analyses of the DEGs using Gene Ontology (GO) and Kyoto Encyclopedia of Genes and Genomes (KEGG) analyses were conducted with the clusterProfiler software package (version 4.12.6) in R [42].

### 4.5. WGCNA

The WGCNA package (version 1.73) in R was used to perform a coexpression analysis of the DEG expression profile with the dynamic branch cutting method [43]. The weight coefficient β should satisfy a correlation coefficient close to 0.8. In this work, β = 17 was selected as the weight coefficient. The network was constructed using blockwise modules to obtain the gene coexpression module (minModuleSize = 30 and Merge Cut Height = 0.25). The correlation coefficient and *p* value between the module’s characteristic vector module eigengene (ME) and the *V. dahliae* fungus cultured at different temperatures were calculated. The coexpression network was visualized using Cytoscape software [44].

### 4.6. qRT-PCR

Based on the cDNA information for each gene of interest, primers were designed in the specific region of the 5′ or 3′ end of the gene sequence using Primer 5.0 software (Table S1). Total RNA was extracted using the RNAprep Pure Polysaccharide and Polyphenol Plant Total RNA Extraction Kit (Tiangen, Beijing, China). The concentration of each RNA sample was determined using a NanoDrop 2000 spectrophotometer (Thermo Fisher Scientific, Waltham, MA, USA). The RNA was reverse transcribed using the M-MLV RTase cDNA Synthesis Kit (TaKaRa, Osaka, Japan) to generate cDNA. Real-time PCR amplification was performed on a Bio-Rad CFX96 Real-time System. An iTaq Universal SYBR Green Supermix (Bio-Rad, Hercules, CA, USA) kit was used, and the method provided was followed, with a total system volume of 20 μL. The reaction procedure was predenaturation at 95 °C for 30 s and 40 cycles of denaturation at 95 °C for 5 s, annealing at 57 °C for 5 s, and extension at 72 °C for 34 s. The results were analyzed by performing a relative quantitative analysis using the 2^–ΔΔCt^ method, and the internal reference gene used was *GhUBQ7* [45].

## 5. Conclusions

Temperature plays an important role in the growth and invasion of *V. dahliae*, which in turn affects the occurrence of *V. dahliae* in cotton. The optimal temperature for the growth of *V. dahliae* Vd-3 hyphae is 25 °C, which is suitable for spore production. RNA-seq revealed that photosynthesis and sugar metabolism processes are essential for cotton defense against *V. dahliae*, and twelve key genes related to cotton defense against *V. dahliae* at different temperatures were identified, including four genes encoding transcription factors. These research results reveal the effects of temperature on the growth and infection of pathogens and potential regulatory pathways, providing theoretical support for the effective prevention and control of *V. dahliae* and temperature regulation. However, the current understanding of this relationship in terms of environmental immunity is still in its early stages, and continued exploration of the potential mechanism of temperature regulation of cotton resistance to Verticillium wilt is needed to provide a reference for *V. dahliae* prevention and control strategies and the breeding of disease-resistant varieties.

## Data Availability

The RNA-seq data presented in the study are deposited in the NCBI repository under accession number PRJNA1147881.

## Supplementary Materials

The following supporting information can be downloaded at: https://www.mdpi.com/article/10.3390/plants13192688/s1, Table S1: All primers used in this study. Table S2: Summary statistics for the RNA-seq analysis of *G. hirsutum*. Figure S1: Scatter plot of the correlations between the RNA-seq data and expression levels determined using qRT-PCR.
