# Supplementary figures and images for: RNA-Seq Analysis and Candidate Gene Mining of Gossypium hirsutum Stressed by Verticillium dahliae Cultured at Different Temperatures
Source: Plants (Basel). 2024 Sep 25;13(19):2688. doi: 10.3390/plants13192688 (PMC11479098; doi:10.3390/plants13192688)

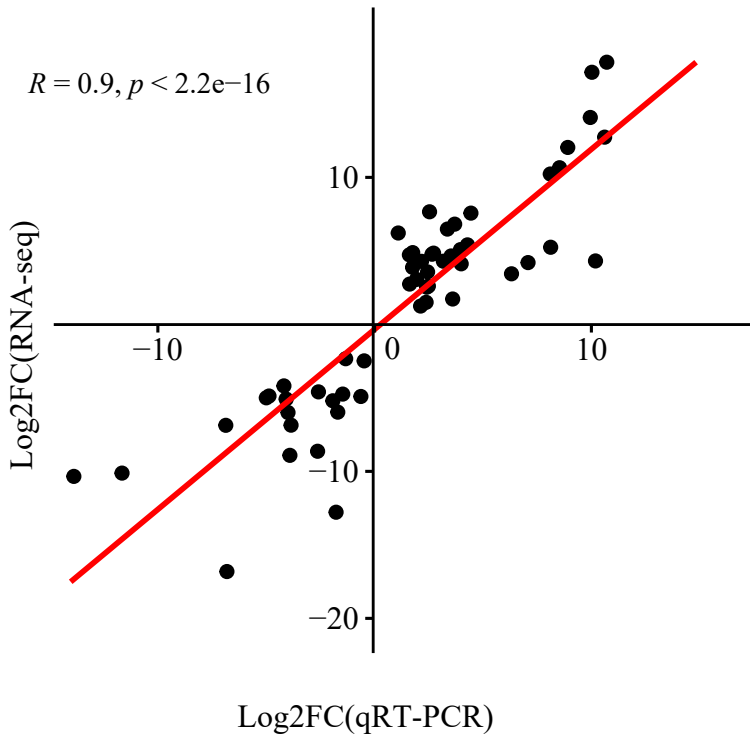

Supplement: Supplementary file 1 [file plants-13-02688-s001.zip › Figure S1.pdf]
